# The Future of British Nursing


**Published:** 1916-09-09

**Authors:** 


### THE MATRONS' AND SISTERS' DEPARTMENT.

### THE FUTURE OF BRITISH NURSING.*

### II.—TRAINING AND CERTIFICATION.

THE holidays have caused a lull in the completion of various technical matters connected with the launching of the College of Nursing, and this has given time to those who will initially have the responsibility of getting things started to realise a few at any rate of the problems which lie ahead. Heretofore the abler the matron and the larger the training-school under her guidance the greater has been the practically uncontrolled responsibility which she has exercised. On the whole this system has worked fairly well, but it has not invariably resulted in a haven of bliss for the women during the period of their training, and it has led to the creation of a feeling, sometimes unconscious but none the less definite, that each of the principal schools, or most of them, are undoubtedly better than their neighbours, and that whatever they may have constituted a practice is the best and often the only one which can be accepted and pursued by matrons of the highest intelligence.

It must be manifest, and indeed it is becoming more widely recognised every week, that, having regard to the ever-increasing demand for trained nurses in this country, and to the capacity of the existing training-schools being able adequately to meet the demand at the conclusion of the war and for financial reasons, it may be necessary to reorganise the whole system of nurse-training throughout Great Britain. Those who recognise these patent facts, and who have imagination enough to forecast the immense value and added strength the College of Nursing should result in, will prepare their minds for these important developments and place themselves in a position to understand and deal with them. They will so equip themselves to co-operate with an open mind having the one desire that every change shall be well thought out, and be established upon the basis best calculated to secure that the British system of nurse-training will be recognised as the best in the world.

It follows from these considerations that several habits in training-school practice which prevail at present must necessarily be modified. There has been a certain amount of competition, which is always wholesome, between training-schools in the past, and some if not most of these habits may depend in some measure upon this fact. It is exceedingly gratifying to a matron of a certain type to be able to keep the probationers for the whole of the three years' training continually under her control and supervision. In many ways at the best schools this practice has become a guarantee of relative efficiency on the part of the fully certificated nurse, but where the school is so large as to render it impossible for the matron to supervise personally the work of each woman undergoing training in every department of the hospital, she necessarily has to depend upon the reports of others, and these reports may be, and sometimes are, prejudiced, so that it may become impossible for the matron to hall-mark every nurse certificated from her school with the stamp of her individual approval, because she is a woman of the highest character and the keenest apprehension.

In the smaller schools the policy of maintaining the habit of providing that every probationer shall throughout the whole three years of her training be directly under the supervision and control of the same matron may result from it. It brings the matron and the probationer into fairly frequent personal contact. It results under the best matrons in the building up of mutual sympathy, and leads to a higher standard of personal effort and personal work. But the policy based upon the habit we are dealing with here may be liable to abuse. In those cases where a certificate is granted after only two years' training, and the nurses are employed on the private staff of the hospital and supplied to the public, for the payment of money, as fully trained nurses, the *morale* of the probationer, as she comes to know and realise what is done, may be seriously affected, and she may have her faith in her hospital seriously shaken by the realisation that she is being farmed out, before she is really certificated, to make money, even supposing attempts are made not to send her to any case except one in the nursing of which she has had experience during her first two years of training.

The feeling of the majority of the wisest leaders of nursing in this country, and of those who have most to do with training nurses, is overwhelmingly and properly opposed to the farming of nurses with two years' training for the benefit of any hospital authorities. A further objection to it is that when a partially trained nurse of two years' standing is sent out to private cases she must necessarily lose the discipline and the check of constant touch with her matron and of those who have trained her. She is left largely on her own resources, and if she fails from want of experience or other cause to give satisfaction, her nursing career may be, in our judgment, unjustly injured, not necessarily from any fault of her own, but from this defect in the system of the school where she entered as a probationer. We have always hoped, and we shall continue to hope, that those who are responsible for the two-years' certificate will, in the interests of nurses and nursing, and of the honour and good name of their hospital, determine on the institution of the College of Nursing to abolish this anomaly. It will be a golden opportunity for them to join hands with the overwhelming majority of British nurse-training schools by instituting the three-years' course before certification.

* The previous article appeared on August 12, p. 455.

